# Exoproteome Analysis of the Seaweed Pathogen *Nautella italica* R11 Reveals Temperature-Dependent Regulation of RTX-Like Proteins

**DOI:** 10.3389/fmicb.2017.01203

**Published:** 2017-06-29

**Authors:** Melissa Gardiner, Adam M. Bournazos, Claudia Maturana-Martinez, Ling Zhong, Suhelen Egan

**Affiliations:** ^1^School of Biological Earth and Environmental Sciences–Centre for Marine Bio-Innovation, The University of New South Wales, Sydney,NSW, Australia; ^2^Bioanalytical Mass Spectrometry Facility, Mark Wainwright Analytical Centre, The University of New South Wales, SydneyNSW, Australia

**Keywords:** Roseobacter, virulence, secretome, marine bacteria, RTX-toxin, macroalgae, symbiosis, proteomics

## Abstract

Climate fluctuations have been linked to an increased prevalence of disease in seaweeds, including the red alga *Delisea pulchra*, which is susceptible to a bleaching disease caused by the bacterium *Nautella italica* R11 under elevated seawater temperatures. To further investigate the role of temperature in the induction of disease by *N. italica* R11, we assessed the effect of temperature on the expression of the extracellular proteome (exoproteome) in this bacterium. Label-free quantitative mass spectrometry was used to identify 207 proteins secreted into supernatant fraction, which is equivalent to 5% of the protein coding genes in the *N. italica* R11 genome. Comparative analysis demonstrated that expression of over 30% of the *N. italica* R11 exoproteome is affected by temperature. The temperature-dependent proteins include traits that could facilitate the ATP-dependent transport of amino acid and carbohydrate, as well as several uncharacterized proteins. Further, potential virulence determinants, including two RTX-like proteins, exhibited significantly higher expression in the exoproteome at the disease inducing temperature of 24°C relative to non-inducing temperature (16°C). This is the first study to demonstrate that temperature has an influence exoproteome expression in a macroalgal pathogen. The results have revealed several temperature regulated candidate virulence factors that may have a role in macroalgal colonization and invasion at elevated sea-surface temperatures, including novel RTX-like proteins.

## Introduction

Seaweeds (macroalgae) are key ecosystem engineers of temperate marine coastal habitats. They provide a protective habitat and nursery for many other species and as primary producers they act as a major food source (Wernberg et al., 2011). Unfortunately these important marine habitat formers are also susceptible to disease as a result of environmental stress and/or microbial pathogens (Gachon et al., 2010; Hollants et al., 2013; Egan et al., 2014). For example the red macroalga *Delisea pulchra* undergoes bleaching of its thallus corresponding to increased sea-surface temperatures (Campbell et al., 2011; Case et al., 2011). A combination of laboratory and field experiments have demonstrated that under increased host stress (such as that imposed by higher temperatures) *D. pulchra* has reduced levels of its natural chemical defense (furanones) (Campbell et al., 2011; Case et al., 2011). This reduced algal defense results in the increased susceptibility to infection by bacterial pathogens, including the Roseobacter species *Nautella italica* R11 (formally *Ruegeria* sp. R11) under the elevated sea-surface temperatures of 24°C (Campbell et al., 2011; Case et al., 2011; Fernandes et al., 2011; Kumar et al., 2016).

Genomic analysis of *N. italica* R11 has provided insight into the potential virulome of the marine pathogen, which includes a range of transporter systems that could facilitate both the uptake of algal metabolites and the secretion of toxins and/or degradative proteins (Fernandes et al., 2011). The *N. italica* R11 genome encodes for several proteins with homology to repeats-in-toxin (RTX) proteins that function as cytotoxins in other pathogens (Linhartova et al., 2010), and these proteins may mediate tissue damage on a *D. pulchra* host (Fernandes et al., 2011). However, with the exception of recent studies demonstrating the role of oxidative stress resistance and quorum sensing-mediated colonization (Gardiner et al., 2015a,b), the relative importance of other virulence traits in *N. italica* R11 remains unknown.

High throughput mass spectrometric techniques permit the identification of the subset of bacterial proteins secreted in the extracellular milieu (the exoproteome) (Desvaux et al., 2009; Christie-Oleza and Armengaud, 2010). Recent data for Roseobacter clade members has highlighted the benefit of exoproteome analysis for the identification of proteins previously overlooked by genomic studies but important for bacterial physiology/host interactions (Zech et al., 2009; Christie-Oleza and Armengaud, 2010; Christie-Oleza et al., 2012b; Kossmehl et al., 2013; Christie-Oleza and Armengaud, 2015). Moreover, exoproteome analysis of human, animal, and plant pathogens has revealed new putative virulence determinants (Madec et al., 2014; Campbell et al., 2015; He et al., 2015; Mandelc and Javornik, 2015), and provided insight into the temperature-dependent regulation of protein secretion (Kimes et al., 2012). However, to date, nothing is known about the exoproteome composition or the influence of temperature on protein expression in seaweed pathogens.

Here, we use label free mass spectrometry to investigate for the first time the exoproteome of the seaweed pathogen *N. italica* R11. As elevated sea-surface temperature plays a key role in the induction of the seaweed bleaching disease by *N. italica* R11, we also compared the exoproteome of *N. italica* R11 exposed to disease (24°C) and non-disease (16°C) inducing temperatures to investigate potential virulence factors that could facilitate pathogenesis in this macroalgal pathogen.

## Materials and Methods

### Bacterial Culture Conditions Used in the Study

*Nautella italica* R11 cultures were routinely grown in half strength (18.4 g/l) marine broth (Difco, Becton Dickson, United States) with shaking (180 rpm) at room temperature (21°C) from cultures stored at -80°C in 10% glycerol stocks. The cultures used for protein analysis were inoculated at 10% v/v using an overnight culture (grown directly from a frozen stock) harvested at an OD600 = 1 and rinsed twice in fresh media before being incubated in 200 ml of media at either 24°C (disease-inducing temperature) or 16°C (non-disease-inducing temperature). Cells were harvested by centrifugation at early stationary phase, as determined using spectrophotometric analysis (Supplementary Figure S1). Early stationary phase was selected for analysis as previous studies investigating the virulence of *N. italica* R11 were conducted using bacteria at this growth phase (Case et al., 2011; Fernandes et al., 2011) and the expression of virulence and secondary metabolites has been linked to stationary growth phase in other marine bacteria (Egan et al., 2002; Vanden Bergh et al., 2013).

### Label-Free Quantitation of the *N. italica* R11 Exoproteome

To obtain the supernatant fraction (exoproteome) of *N. italica* R11 cells, 200 ml of culture was treated with protease inhibitor cocktail (Sigma) before filtration through a 0.2 μm Millex syringe filter unit (Merck Millipore). Peptides ≥ 3 kDa were then obtained by centrifugation through an Amicon Ultra-4 unit (Merck Millipore) according to the manufacturer’s instructions. The concentration of the eluted proteins was determined using a 2-D Quant Kit (GE Healthcare) according to the manufacturer’s instructions.

The supernatant proteins were separated and digested in-gel using SDS-PAGE due to the presence of low molecular weight peptides from the cell growth media (Additional file, Supplementary Figure S2). Fifteen micrograms of protein were denatured as described previously (Burgos-Portugal et al., 2012), separated on an any kD^TM^ Mini-PROTEAN^®^ TGX^TM^ Precast Gel (Bio-Rad) by electrophoresis for 1.5 h at 100 V and stained using SimplyBlue^TM^ SafeStain (Invitrogen). Once visualized the proteins on the SDS-PAGE gel were cut into three sections (Supplementary Figure S2), placed into three microfuge tubes, and destained (with 25% acetonitrile/25% NH_4_HCO_3_). Fifty microliters of the reducing agent (10 mM DTT, 50 mM NH_4_HCO_3_) was added before incubation at 37°C for 30 min. The reducing agent was removed and 50 μl of the cysteine-blocking reagent (200 mM Iodoacetamide, 100 mM NH_4_HCO_3_) was added before incubated at 37°C for 30 min. The gel pieces were dehydrated by adding 100% acetonitrile to each tube. The acetonitrile was then removed and 60 μl of 2 ng/μl trypsin in 20 mM NH_4_HCO_3_ was added to digest the proteins at 37°C overnight. The digested peptides were then solubilized in 1% formic acid, 0.05 HFBA (heptafluorobutyric acid) and the peptides (corresponding to the three gel slices) were pooled. Digested peptides were separated by nano-LC using an Ultimate 3000 HPLC and autosampler system (Dionex, Amsterdam, Netherlands) as described previously (Kaakoush et al., 2015). High voltage (2000 V) was applied to low volume tee (Upchurch Scientific) and the column tip positioned ∼0.5 cm from the heated capillary (*T* = 280°C) of an Orbitrap Velos (Thermo Electron, Bremen, Germany) mass spectrometer. Positive ions were generated by electrospray and the Orbitrap operated in data dependent acquisition mode (DDA). A survey scan m/z 350-1750 was acquired in the Orbitrap (Resolution = 30,000 at m/z 400, with an accumulation target value of 1,000,000 ions) with lockmass enabled. Up to the 10 most abundant ions (>5,000 counts) with charge states > +2 were sequentially isolated and fragmented within the linear ion trap using collisionally induced dissociation with an activation *q* = 0.25 and activation time of 30 ms at a target value of 30,000 ions. M/z ratios selected for tandem mass spectrometry (MS/MS) were dynamically excluded for 30 s. Mass spectrometry peak lists were generated using Mascot Daemon/extract_msn (Matrix Science) using the default parameters and a decoy false discovery rate of *p*-value < 0.01, and were submitted to the database search program Mascot (version 2.1). Mascot determined the peptides with ion score cut-off at 20. Search parameters were: precursor tolerance 4 ppm and product ion tolerances ± 0.4 Da, alkylation of cysteine as fixed modification, methionine oxidation as variable modification, enzyme specificity was trypsin, 1 missed cleavage was possible, and the NCBI non-redundant (nr) database searched (accessed May 2009). Each MS/MS spectrum was compared to an in-house *N. italica* R11 genome (available via IMG, Genome ID 647533206) (Markowitz et al., 2006). Three technical replicates were performed for each biological replicate.

### Analysis of the Exoproteome Data

Progenesis^®^ QI for Proteomics (version 2.0, Nonlinear Dynamics, United Kingdom) was used to analyze the mass spectrometry data. The acquired spectra were loaded into the Progenesis^®^ software and the ion intensities of six runs (three replicates at 16°C, three at 24°C) were examined and label-free quantification was performed. Replicate 1 for temperature condition 16°C was chosen as the reference and the retention times of all six samples were aligned. Features with only one charge and over four changes were excluded from further analysis. Samples were grouped to their experimental condition (16°C versus 24°C). One-way analysis of variance (ANOVA) statistical analysis was performed using transformed normalized abundances. Only proteins represented by two or more peptides, including at least one unique peptide, where considered in the exoproteome analysis. Proteins with a fold change ± 2 and *p*-value < 0.05 were considered significantly differentially expressed across the two temperature conditions. Identified *N. italica* R11 proteins are described as GenBank accession numbers (Wheeler et al., 2008) and assignment of clusters of orthologous groups (COG) was performed using the IMG/ER database (Markowitz et al., 2006). The predicted exoproteome was determined using the automated SignalP 3.0 analysis (Bendtsen et al., 2004) of the *N. italica* R11 genome in IMG/ER and by manually searching the genome for proteins containing the TAT (twin-arginine translocation) pathway signal sequence [TIGRfam family (TIGR01409)] in the translated nucleotide sequence (Berks et al., 2000). Supernatant proteins without a characterized signal peptide were scanned using the SecretomeP 2.0 sever (Bendtsen et al., 2005), with a SecP score > 0.5 considered as evidence of non-classical protein secretion. The subcellular localization (SCL) of the supernatant proteins was assessed using the open source web-based predictor tool, PSORTb version 3.0 (Yu et al., 2010). Proteins of interest were scanned for protein families against the Integrated resource of protein families, domains and functional sites (InterPro) database (Mulder et al., 2007).

## Results and Discussion

Evidence suggests that environmental perturbations, including increased seawater temperatures, contribute to the susceptibility of macroalgae to microbial disease (Gachon et al., 2010; Campbell et al., 2011; Koch et al., 2013). Therefore, an understanding of the impact of increased temperature on the virulence of bacterial seaweed pathogens is important, particularly within temperate ecosystems that are vulnerable to warming ocean currents (Wernberg et al., 2011; Egan et al., 2014). Here, we examined the influence of temperature on the exoproteome of the seaweed pathogen *N. italica* R11 using label-free LC-MS/MS.

### The Exoproteome of *N. italica* R11

**FIGURE 1:**
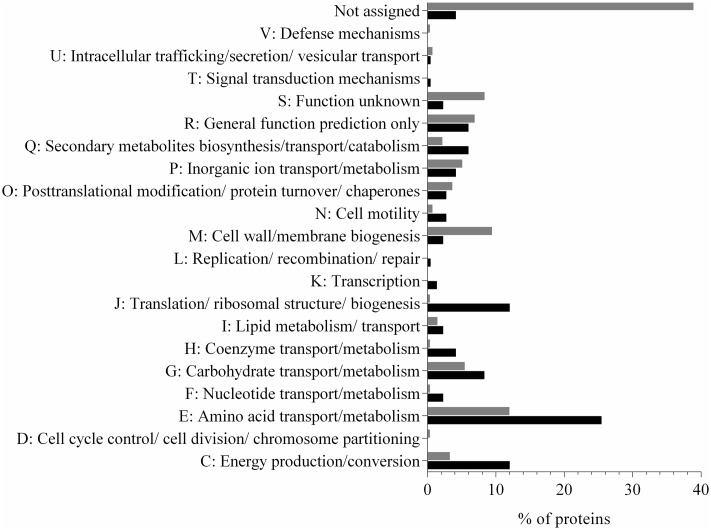
Distribution of protein COG categories in the predicted secretome (gray) and detected (black) exoproteome of *N. italica* R11. The percentage of proteins for each COG category is shown. ‘Not assigned’ denotes proteins that have not been assigned to a COG group in IMG/ER. Proteins were detected in three biological replicates at either 24°C or 16°C (Supplementary Table S1).

In this study, we identified 207 proteins in the supernatant fraction of *N. italica* R11 (Supplementary Table S1), which corresponds to over 5% of the protein coding genes in the genome. The most abundant protein detected in the supernatant fraction (at >27%) was a flagellin domain protein (EEB70216); all other proteins had an individual abundance of <5% (Supplementary Table S1). Twenty-two percent of the predicted exoproteome [annotated based on the presence of a TAT or Sec signal using IMG/ER (Markowitz et al., 2006)] was identified in the supernatant of *N. italica* R11. While many (16%) of the supernatant proteins with a signal peptide were predicted by PSORTb *in silico* analysis to be located in the periplasm (Yu et al., 2010), 16 proteins had a unknown cellular location and 12 proteins were identified as extracellular factors in agreement with the mass spectrometry data. Of the proteins that were predicted to be secreted but were not identified in the mass spectrometry analysis, many (116) proteins are uncharacterized factors [assigned to either COG S or no COG category (NA), **Figure 1**], and it is possible that these proteins may be expressed by *N. italica* R11 under growth conditions not tested here, for example, when directly associated with a *D. pulchra* host.

In contrast, a larger proportion of proteins associated with amino acid metabolism (COG E), protein translation (COG J) and energy production (COG C) were detected in the exoproteome of *N. italica* R11 than had been predicted from genome data (**Figure 1**). PSORTb analysis of the supernatant proteins suggested that at least 62% are localized in the cytoplasm, including, for example, ribosomal proteins (e.g., GenBank: EEB71264, EEB70057) and enzymes involved in the tricarboxylic acid (TCA) cycle (e.g., GenBank: EEB69552, EEB69852) (Supplementary Table S1). These typically cytosolic proteins are included in the list of detected supernatant proteins, however, they are not considered to be part of the ‘true exoproteome’ of this bacterium. Instead these proteins were likely introduced into the supernatant by cell lysis during sample processing as has been previously reported for bacterial exoproteome data (Wang et al., 2013; Gotz et al., 2015). Future work may utilize subcellular fractionation to fully resolve the cellular location of putative virulence factors in *N. italica* R11 under the disease-inducing temperature.

Other studies have demonstrated that bacterial exoproteomes commonly contain a high proportion of proteins that do not possess a Sec or TAT signal sequence (Christie-Oleza and Armengaud, 2010; Kaakoush et al., 2010; Zijnge et al., 2012). For example in the Roseobacter clade bacterium *R. pomeroyi* DSS-3, 65% of exoproteome proteins did not have a signal peptide (Christie-Oleza and Armengaud, 2010). In well characterized pathogenic bacteria proteins such as antioxidant and metabolic enzymes have been observed to be secreted into the extracellular mileu via non-classical pathways (Bendtsen et al., 2005; Emanuelsson et al., 2007; Wang et al., 2016). Over ten percent of the proteins detected in the *N. italica* R11 exoproteome were predicted by SecretomeP analysis to be secreted via a non-classical pathway. These include two enzymes, pyruvate dehydrogenase (EEB70022) and glyceraldehyde-3-phosphate dehydrogenase (EEB70882), which are best known for their role as cytoplasmic glycolytic enzymes and were localized to the cytoplasm by PSORT analysis (Yu et al., 2010). Interestingly both of these proteins have been reported in Gram-positive bacteria to be non-classically secreted (Wang et al., 2016) and shown to act as cell surface adhesions and for iron sequestration in a number of bacterial pathogens (Modun et al., 2000; Egea et al., 2007; Chauhan et al., 2015). Experimental investigation of the subcellular localization and membrane transport of proteins would provide insight into the role of non-classically secreted proteins in the physiology of *N. italica* R11, as has been modeled for the Roseobacter species *Phaeobacter inhibens* DSM 17395 (Kossmehl et al., 2013).

Numerous proteins associated with the transport of amino acids (e.g., GenBank: EEB71458), carbohydrates (e.g., GenBank: EEB69824), phosphates (e.g., GenBank: EEB70646), secondary metabolites (e.g., GenBank: EEB71180) and yet uncharacterized factors (e.g., GenBank: EEB69742) were detected in the *N. italica* R11 exoproteome (**Figure 1** and Supplementary Table S1). This finding is in line with previous suggestions that this bacterium is proficient in assimilating metabolites from the environment (Fernandes et al., 2011). Fifteen percent of the proteins detected in the exoproteome data for *N. italica* R11 are homologous to bacterial Type I and Type II transporter proteins, indicating that ATP-dependent transport plays a key role in the physiology and metabolism of this pathogenic bacterium. Overrepresentation of Type I and II secretion systems has previously been reported for Roseobacter clade members, including *Ruegeria pomeroyi* DSS-3 and *Phaeobacter* DSM 17395 (Christie-Oleza and Armengaud, 2010; Christie-Oleza et al., 2012b; Durighello et al., 2014), where the abundance of specialized transport systems is similarly hypothesized to facilitate the assimilation of a diverse range of low concentration substrates, including metabolites, from host organisms (Christie-Oleza and Armengaud, 2010; Christie-Oleza et al., 2012a; Durighello et al., 2014).

### Putative Temperature-Dependent Virulence Factors Secreted by *N. italica* R11

**FIGURE 2:**
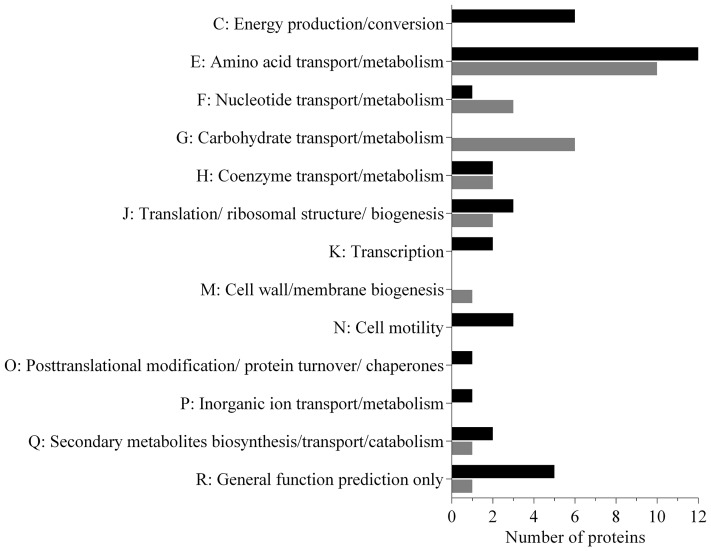
Functional properties of the secretome proteins differentially expressed under disease inducing temperatures in *N. italica* R11. The number of proteins up-regulated (black) and down-regulated (gray) for each COG category is given. Sixty-three proteins were found to be differentially expressed in the supernatant; 37 proteins were up-regulated, and 26 were down-regulated (Supplementary Table S2).

Analysis of the data revealed that a subset (30%) of the *N. italica* R11 exoproteome was differentially expressed in cells at 24°C relative to those grown at 16°C (**Figure 2** and Supplementary Table S2). The temperature-dependent secreted proteins are associated with transport, biogenesis and virulence related functions (**Figure 2**: COG E, G, J, Q), and numerous proteins (15%) are annotated as factors that mediate the binding and transport of a range of substrates including carbohydrates (e.g., GenBank: EEB71800) and amino acids (e.g., GenBank: EEB71544) (Supplementary Table S2). Many of these transport factors were down-regulated at 24°C, including a leucine-binding (InterPro: IPR028081) extracellular receptor protein (GenBank: EEB71945) that was three-fold down-regulated. These data suggest that *N. italica* R11 modulates the uptake and/or transport of a range of metabolites and nutrients from an algal host or the surrounding environment in response to temperature conditions.

The exoproteome of *N. italica* R11 up-regulated at 24°C also includes factors potentially involved in motility, specifically, two flagella hook proteins (GenBank: EEB71275, EEB70112) (**Figure 2**, COG N), one protein containing the flagellar hook-length control domain (GenBank: EEB71644) and one putative flagella-associated protein (GenBank: EEB72697) (Supplementary Table S2). Motility has previously been suggested as a putative virulence determinant for *N. italica* R11 (Fernandes et al., 2011; Egan et al., 2014), and flagella hook proteins orthologous to those over-represented in the *N. italica* R11 exoproteome have a key role in the virulence of other bacterial pathogens (Mariappan et al., 2011; Haiko and Westerlund-Wikström, 2013). Increased abundance of flagella hook proteins in the exoproteome at 24°C could suggest that temperature positively effects motility in this bacterium, and future work should further investigate the link between motility, temperature, and virulence gene expression in *N. italica* R11.

Several of the proteins up-regulated in the supernatant of *N. italica* R11 under disease-inducing temperatures have a general predicted function only (GenBank: EEB72449, EEB72375, EEB72304, EEB72352) (Supplementary Table S2) (**Figure 2**: COG R) and thus require further investigation to elucidate their precise function in *N. italica* R11. For example, a putative zinc-dependent M16 peptidase domain (pfam00675) protein (GenBank: EEB72304) (Supplementary Table S2) that was up-regulated two-fold at 24°C has homology to an uncharacterized family of (insulinase-like) metallopeptidases that are hypothesized to be involved in the degradation of small polypeptides (Fricke et al., 1995).

### *N. italica* R11 Secretes Two RTX-Like Toxins under Disease-Inducing Temperatures

The data generated in this study shows that *N. italica* secretes at least eight proteins that possess a hemolysin-type calcium-binding domain (COG Q: COG2931) characteristic of RTX proteins (GenBank: EEB69413, EEB69465, EEB69635, EEB69729, EEB71736, EEB70003, EEB70215, EEB71599) (Supplementary Table S2) (Linhartova et al., 2010). Together these eight proteins comprise over 5% of the detected exoproteome of *N. italica* R11 at 24°C (Supplementary Table S1). RTX proteins encompass a range of proteins, including leukotoxins, adhesins, and proteases (Linhartova et al., 2010) and have been well studied in other bacterial pathogens as virulence factors that mediate colonization, invasion and host damage (Lee et al., 2008; Li et al., 2008; Vigil et al., 2012; Wiles and Mulvey, 2013). While the *N. italica* R11 RTX-like proteins possess the RTX toxins related domain (COG2931), the proteins exhibit no significant sequence homology (<25%) to RTX proteins characterized in pathogenic bacteria (Linhartova et al., 2010; Wiles and Mulvey, 2013). Further, unlike well-characterized examples of RTX proteins that possess a Sec signal peptide (Linhartova et al., 2010), the *N. italica* R11 RTX-like proteins are predicted to be secreted via a non-classical system (SecP scores provided in Supplementary Table S1) (Bendtsen et al., 2005). Secretion of atypical RTX-like proteins has been demonstrated for Roseobacter clade members related to *N. italica* R11 (Christie-Oleza et al., 2012b; Durighello et al., 2014). This method of secretion has been proposed for the highly abundant RTX-like proteins in *Phaeobacter* strain DSM 17395 and *Ruegeria pomeroyi* DSS-3 with each protein comprising over half of the total exoproteome in these bacteria (Christie-Oleza and Armengaud, 2010; Durighello et al., 2014). Despite the significance of the atypical RTX-like proteins in the exoproteomes of Roseobacter species, the precise biological roles of these proteins have not yet been elucidated.

**FIGURE 3:**
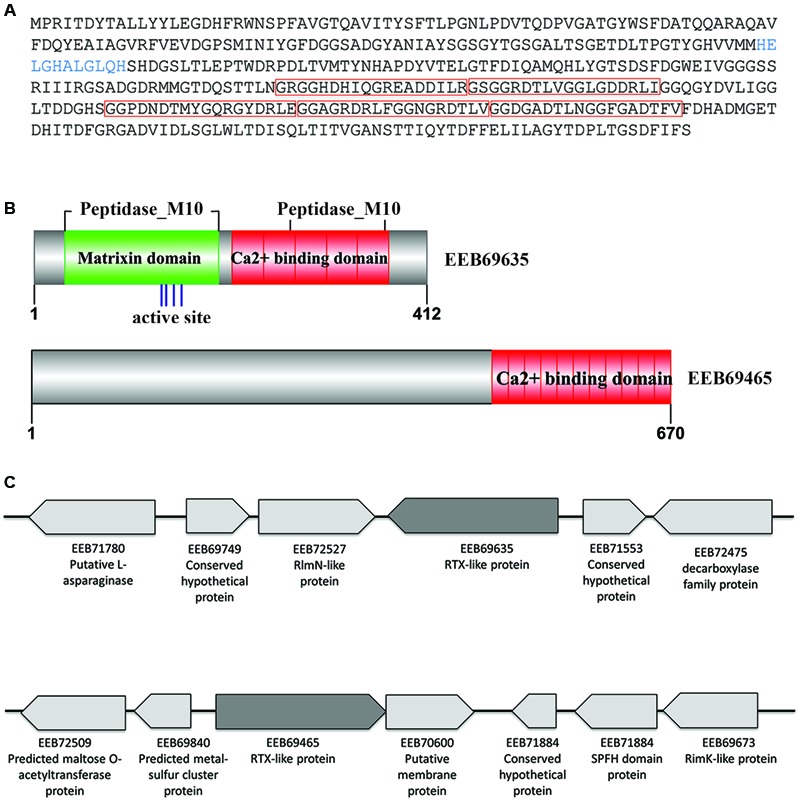
Characteristics of the *N. italica* R11 RTX-like proteins secreted under disease inducing temperatures. **(A)** Translated amino acid sequence of EEB69635. The HExxH zinc-binding site/active site is shown in blue. The predicted protein sequence contains five calcium-binding repeat domains (pfam00353 HemolysinCabind domain) as indicated by red boxes. **(B)** Conserved domain structure of the RTX-like proteins EEB69635 and EEB69465, respectively. The zinc binding site/active site of EEB69635 is indicated by blue lines, with the HemolysinCabind domains in red and the matrixin domain (pfam00413) in green. **(C)** Genomic context of RTX-like toxins (EEB69635 and EEB69465 in dark gray) showing neighboring genes (not to scale); GenBank accession numbers and protein prediction are indicated.

Two of the *N. italica* R11 RTX-like proteins were significantly up-regulated in the exoproteome under disease inducing temperatures (GenBank: EEB69465, EEB69635) (**Figure 3** and Supplementary Table S2). To the best of the authors’ knowledge, this constitutes the first evidence for temperature-regulation of RTX-like proteins in a Roseobacter clade member. The *N. italica* R11 RTX-like protein with the largest fold increase in the exoproteome [i.e., 5.5-fold up-regulated at the disease-inducing temperature (Supplementary Table S2)] was EEB69635. Phylogenetic analyses with homologous sequences found EEB69635 clustered with uncharacterized RTX-like proteins derived from other Roseobacters and was distantly related to characterized RTX-proteins, such as the serralysin protein zapA from *Proteus mirabilis* (Q11137) (Supplementary Figure S3A). Analysis of the domain structure of EEB69635 revealed that in addition to containing the Ca^2+^ binding protein RTX toxin-related domain (COG2931) typical for RTX-toxins, EEB69635 possesses an N-terminal peptidase_M10 domain (Pfam domain: pfam00413) with a metal-binding HEXXH motif, characteristic of metalloproteases related to eukaryotic matrixins that degrade components of the extracellular matrix (Visse and Nagase, 2003). EEB69635 also has a peptidase_M10_C serralysin-like C-terminal domain (Pfam domain: pfam08548). This C-terminal pfam08548 domain forms a “corkscrew” structure that is predicted to be important for protein secretion. While the M10 peptidase domain is yet to be fully characterized for bacterial proteins, it is interesting to hypothesize that the RTX-like protein EEB69635 may function in *N. italica* R11 to aid in the degradation of algal host tissue. Analysis of neighborhood genes (**Figure 3**) indicates EEB69635 is not located within a larger operon and annotation of surrounding genes does not provide any further functional insights of this protein.

The second differentially expressed RTX-like protein (EEB69465) was over three-fold upregulated (Supplementary Table S2) and while this protein contains a Ca^2+^ binding protein RTX toxin-related domain (COG2931) at the C-terminus, there is no evidence of sequence homology at the N-terminus to other characterized domains structures (**Figure 3**). Phylogenetic analyses with homologous sequences showed that EB69465 clustered closely with similar uncharacterized RTX-like proteins from related Roseobacter species (Supplementary Figure S3B), thus did not provide further insight into its function. Directly downstream of the gene encoding for EEB69465 is a putative membrane protein and these two genes are likely to be encoded within a single operon. However, as with EEB69635, the gene neighborhood of EEB69465 provides little information related to the function of this protein. It is therefore difficult to speculate a specific role for EEB69465 in the pathogenesis of *N. italica* R11 beyond a predicted calcium binding function. Future work should elucidate the molecular function of these temperature-regulated RTX-like proteins in the pathogenesis and/or physiology of *N. italica* R11.

## Conclusion

This study is the first to demonstrate that *N. italica* R11 modulates the expression of a subset of its exoproteome in response to temperature, and it provides the foundation for future investigations into the function of the temperature-dependent secreted proteins in the pathogenicity and/or environmental persistence of *N. italica* R11. Further studies using RNA-seq techniques maybe undertaken in the future to assess virulence expression in this pathogen, for example, by characterizing transcription when the bacterium is exposed to host metabolites or exudates. In this study, the proteins that were most highly secreted from *N. italica* R11 under disease-inducing temperature, including the RTX-like proteins, constitute novel virulence factors that may play an important role in the colonization and bleaching of *D. pulchra* cells. While future studies are required to verify the expression levels and subcellular location of the proteins identified here, this foundational work highlights the potential importance of temperature for the expression of virulence factors in the macroalgal pathogen, *N. italica* R11. A relevant finding, given that increasing ocean temperatures and climate change are predicted to cause greater host stress and more extensive disease events in macroalgae.

## Author Contributions

MG participated in the study design, contributed to the data analysis and wrote the manuscript. AB completed the protein experiments and data analysis, and drafted parts of the manuscript. CM-M participated in the data analysis and drafted parts of the manuscript. LZ participated in the study design and performed the protein analysis. SE conceived the study, and participated in the study design and drafting of the manuscript. All authors read and approved the final manuscript.

## Conflict of Interest Statement

The authors declare that the research was conducted in the absence of any commercial or financial relationships that could be construed as a potential conflict of interest.
