# Supplementary material for: Exoproteome Analysis of the Seaweed Pathogen Nautella italica R11 Reveals Temperature-Dependent Regulation of RTX-Like Proteins
Source: Front Microbiol. 2017 Jun 29;8:1203. doi: 10.3389/fmicb.2017.01203 (PMC5489592; doi:10.3389/fmicb.2017.01203)
Supplement: Supplementary file 2 [file Table_2.PDF]

Supplementary Table S2: Proteins in the supernatant fraction that were differentially expressed in *Nautella italica* R11 cells grown at the disease-inducing temperature. The proteins are listed using the corresponding GenBank accession number. Fold change (FC) denotes the expression of the protein at 24°C relative to those grown at 16°C, as determined using Progenesis® QI software, with $FC \geq \pm 2$, two or more peptides (at least one unique) and $p < 0.05$ (ANOVA) in all three biological replicates considered significant. COG denotes the clusters of orthologous groups (COG) category assigned to each protein. Signal peptide indicates whether the protein contains a predicted secretory signal peptide assigned to the either the general secretory pathway (Sec) (Bendtsen et al., 2004), twin-arginine translocase pathway (Tat) (Berks et al., 2000), or a non-classical pathway (NCP) (Bendtsen et al., 2005). N indicates that no signal peptide was predicted for the protein. Abundance denotes the average normalized abundance of each protein at either 16°C or 24°C, as calculated by the Progenesis® software.

GenBank accession	Functional Protein Prediction	FC	COG	Signal Peptide	Abundance at 16°C	Abundance at 24°C
EEB69263	extracellular ligand-binding receptor	-9.79	E	Sec	187000.00	19100.00
EEB69272	ABC-type xylose transporter, D-xylose-binding protein	-4.47	G	Sec	161000.00	35900.00
EEB69273	ABC-type oligopeptide/dipeptide transporter, periplasmic substrate-binding protein	-2.69	E	Sec	248000.00	92400.00
EEB69413	Rhizobiocin	-9.88	Q	NCP	10400.00	1047.26
EEB69436	anthranilate phosphoribosyltransferase	3.45	E	N	5298.11	1537.21
EEB69465	RTX-like protein	3.01	Q	NCP	7920.41	23800.00
EEB69532	uroporphyrinogen decarboxylase	5.6	H	N	9018.47	1609.51
EEB69635	RTX-like protein	5.55	Q	NCP	2470.43	13700.00
EEB69686	iron-containing alcohol dehydrogenase	2.18	C	N	28300.00	13000.00
EEB69706	aspartate-semialdehyde dehydrogenase	-4.65	E	N	9530.72	2051.44
EEB69791	glutamyl-tRNA synthetase	2.71	J	N	11300.00	4146.12
EEB69943	lysyl-tRNA synthetase	2.36	J	N	26400.00	11200.00
EEB69948	phosphopyruvate hydratase	-3	G	N	16300.00	5444.68
EEB70015	glutathione S-transferase	-3.87	J	N	38200.00	9852.36
EEB70112	flagellar hook-associated protein FlgK	3.08	N	N	53700.00	166000.00
EEB70167	succinyl-CoA synthetase (ADP-forming) alpha subunit	2.29	C	N	37200.00	16200.00
EEB70168	secretion protein HlyD	-3.27	M	N	25600.00	7810.53
EEB70266	3-isopropylmalate dehydrogenase	2.19	CE	N	23600.00	10800.00
EEB70333	adenosylhomocysteinase	-2.62	H	N	19300.00	7372.01
EEB70545	isocitrate dehydrogenase, NADP-dependent	2.07	C	N	58800.00	28400.00
EEB70553	cysteine desulfurase	4.93	E	N	5718.27	1159.27
EEB70605	ABC-type oligopeptide/dipeptide transporter, periplasmic substrate-binding protein	-4.06	E	N	14800.00	3656.18

EEB70617	ABC-type peptide/nickel/opine uptake transporter, periplasmic substrate-binding protein	-2.62	E	TAT-TIGR01409	55600.00	21300.00
EEB70884	glycine cleavage system T protein	-2.63	E	N	15800.00	6010.99
EEB71027	ser/thr protein phosphatase family protein	-2.38	F	N	79200.00	33300.00
EEB71179	S-(hydroxymethyl)glutathione dehydrogenase/class III alcohol dehydrogenase	2.05	R	N	42000.00	20400.00
EEB71221	TRAP-type transport system, TAT pathway signal protein	4.96	E	Sec	43600.00	8784.01
EEB71249	aldehyde dehydrogenase family protein	-2.85	G	N	33100.00	11600.00
EEB71275	flagellar hook-associated protein FlgL family protein	4.15	N	N	14900.00	61900.00
EEB71283	anthranilate synthase, component I	3.51	EH	N	6400.54	1824.57
EEB71315	peptidase M24	2.5	E	N	6817.85	2729.53
EEB71533	malate dehydrogenase	2.22	C	N	41400.00	18700.00
EEB71544	ABC-type branched-chain amino acid transporter, periplasmic branched-chain amino acid-binding protein	3.89	E	Sec	13400.00	3435.18
EEB71644	flagellar hook-length control (FliK) domain protein	6.06	N	NCP	13200.00	80200.00
EEB71685	succinyl-CoA synthetase (ADP-forming) beta subunit	2.18	C	N	95300.00	43700.00
EEB71788	3-deoxy-7-phosphoheptulonate synthase	3.3	E	N	4729.84	1431.23
EEB71800	extracellular solute-binding protein, family 1	-3.2	G	Sec	155000.00	48400.00
EEB71838	bifunctional purine biosynthesis protein PurH	4.15	F	N	8181.41	1973.25
EEB71868	fructose-bisphosphate aldolase class-I	-3.3	G	N	30300.00	9168.26
EEB71891	5-nucleotidase	-2.29	F	Sec	23000.00	10100.00
EEB71895	glutamyl-tRNA synthetase	2.7	J	N	8971.77	3318.31
EEB71945	extracellular ligand-binding receptor	-3.1	E	NCP	40500.00	13100.00
EEB71946	riboflavin biosynthesis protein ribAB	-2.82	H	N	27500.00	9771.03
EEB71985	aminotransferase class-III	2.82	E	N	24100.00	8533.27

EEB72013	extracellular solute-binding protein, family 5	-2.8	E	Sec	48000.00	17100.00
EEB72065	aromatic amino acid aminotransferase apoenzyme	-2.81	E	N	28600.00	10200.00
EEB72079	cytosol aminopeptidase	-4.62	E	N	14100.00	3059.00
EEB72149	fructose-1,6-bisphosphatase, class II	-2.33	G	N	14800.00	6336.32
EEB72203	2,3,4,5-tetrahydropyridine-2,6-dicarboxylate N-succinyltransferase	3.29	E	N	24000.00	7274.37
EEB72304	zinc-dependent peptidase family protein	2.39	R	N	3134.60	1310.40
EEB72352	basic membrane lipoprotein	-2.48	R	Sec	159000.00	64300.00
EEB72375	peptidase U62, modulator of DNA gyrase	2.12	R	Sec	14000.00	6592.65
EEB72449	amidohydrolase family protein	2.16	R	N	5112.07	2364.33
EEB72541	phosphonate metabolism protein PhnM	20.15	P	N	5105.21	253.38
EEB72547	ABC-type branched-chain amino acid transporter, periplasmic substrate-binding protein	2.15	E	Sec	47500.00	22100.00
EEB72572	transcriptional regulator, LysR family	2.96	K	N	5941.39	2007.29
EEB72608	chaperonin HslO	4.42	O	N	5199.53	1176.02
EEB72622	tellurite resistance protein	2.03	L	N	7253.18	3564.33
EEB72625	arginyl-tRNA synthetase	-2.55	J	N	39600.00	15500.00
EEB72647	methionine gamma-lyase	2.42	E	N	15000.00	6189.84
EEB72697	flagellar protein, putative	2.47	R	N	120000.00	296000.00
EEB72720	formate-tetrahydrofolate ligase	-3.56	F	N	76000.00	21300.00
EEB72813	transcriptional regulator, AraC family	16.74	K	N	5013.06	299.47
