# Supplementary material for: Exoproteome Analysis of the Seaweed Pathogen Nautella italica R11 Reveals Temperature-Dependent Regulation of RTX-Like Proteins
Source: Front Microbiol. 2017 Jun 29;8:1203. doi: 10.3389/fmicb.2017.01203 (PMC5489592; doi:10.3389/fmicb.2017.01203)
Supplement: Supplementary file 3 [file Data_Sheet_1.DOCX]

**Additional file**

**Additional Methods**

Growth curves

Growth curve experiments of *Nautella italica* R11 were performed at 16°C and 24°C in ½ marine broth (MB) (Difco™ Marine Broth 2216) to determine the growth conditions required to harvest equivalent cell densities of early stationary phase cells. An overnight culture of *N. italica* R11 grown at room temperature was harvested at an OD600 = 1, rinsed twice in fresh media and 250μl was used to inoculate triplicate flasks containing 25 ml of ½ MB. The cultures were grown at either 16°C or 24°C with agitation at 180 rpm, and cell growth was monitored by determining the OD600 at regular intervals. The entire experiment was repeated three independent times.

Phylogenetic analyses of RTX-like proteins from *N. italica* R11

Bacterial genomes were searched for orthologs to the *N. italica* R11 RTX-like proteins EEB69635 or EEB69465 using a blastp search tool of both the UniProt database (<http://www.uniprot.org/>) and the non-redundant database of the National Centre for Biotechnology Information (NCBI) in May 2017 (Altschul *et al.*, 1990). Orthologs with greater than 35% amino acid sequence identity to either of the two RTX-like proteins from *N. italica* R11 were aligned with ClustalX using the default parameters (Larkin *et al.*, 2007) and the resulting alignments curated with Gblocks to remove gap positions (Talavera and Castresana, 2007). The resulting alignment of 282 and 316 and amino acid positions for EEB69635 or EEB69465 respectively were then subject to maximum-likelihood analysis using PhyML 3.0 with the default LG substitution model and 100 bootstraps (Criscuolo, 2011). Trees were visualised using Dendroscope (Huson and Scornavacca, 2012).

**Additional Results**





**Supplementary Figure S1:** Growth curves of *N. italica* R11 in ½ MB at 16°C (A), or the disease-inducing temperature of 24°C (B). Growth was determined using spectrophotometric determination of optical density (absorbance at 600 nm). Bacterial cells were harvested for proteomics analysis at stationary phase, as indicated by arrows for the respective temperature conditions.

**
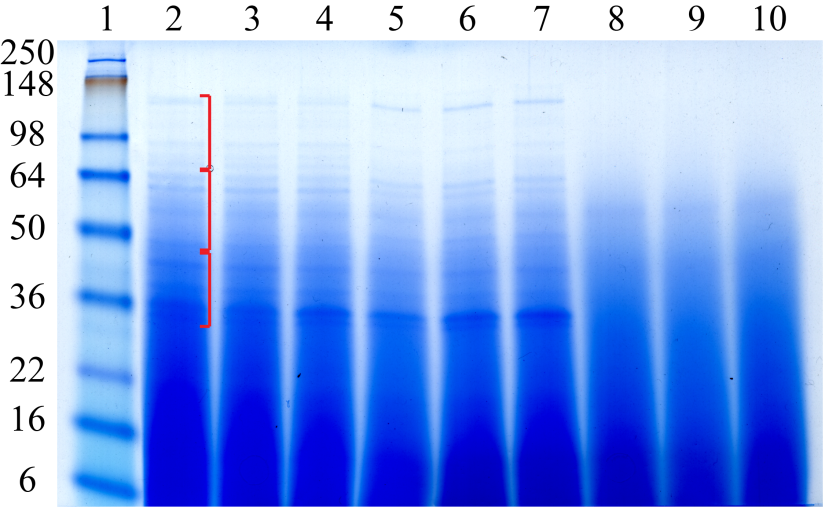
**

**Supplementary Figure S2:** SDS-PAGE of *N. italica* R11 supernatant proteins (lanes 2-4, replicate samples for cultures grown at 16°C; lanes 5-7, replicate 24°C samples; lanes 8-10, media control samples. Lane 1 contains 10 μl SeeBlue® Plus2 Pre-Stained Standard (LC5925, Invitrogen). The media controls were processed identically to the supernatant fraction (see materials and methods). In-gel digestion was used to remove the low molecular weight media peptides from the supernatant proteins, with three gel slices taken as indicated by the red bracket. Once digested, the peptides from the three gel slices were pooled and subjected to MS analysis.

**Figure S3**

**Supplementary Figure S3:** Maximum-likelihood tree of EEB69635 (A) and EEB69465 (B) and orthologous protein sequences. NCBI GenBank accession numbers for each protein sequence is provided in brackets. Bootstrap values for 100 replicates are shown for each node. The scale bar represents 10% sequence divergence.

*In excel spreadsheet*

**Supplementary Table S1:** Proteins identified within the supernatant fraction of *N. italica* R11 within this experiment using two or more peptides (at least one unique). The proteins are listed using the corresponding GenBank accession number. Fold change (FC) denotes the expression of the protein at 24°C relative to those grown at 16°C, with significance assessed using an ANOVA. COG denotes the clusters of orthologous groups (COG) category assigned to each protein. Signal peptide indicates whether the protein contains a predicted secretory signal peptide assigned to the either the general secretory pathway (Sec) [29], twin-arginine translocase pathway (Tat) [30], or a non-classical pathway (NCP) [31]. N indicates that no signal peptide was predicted for the protein. The average (av.) normalized abundance of each protein at either 16°C or 24°C is given, as calculated using Progenesis® QI software. The predicted subcellular location of the proteins was predicted using PSORTb v3.0. Abundance denotes the average normalized abundance of each protein at either 16°C or 24°C, as calculated using Progenesis® QI software. The percentage contribution of each protein to the supernatant fraction is also provided for both temperatures, with the black circle indicating an abundance of 0-0.1%; grey, > 0.1%; pink, >1%; and red >10%.

**References**

Altschul, S.F., Gish, W., Miller, W., Myers, E.W., and Lipman, D.J. (1990). Basic local alignment search tool. *Journal of Molecular Biology* 215(3)**,** 403-410. doi: 10.1016/s0022-2836(05)80360-2.

Criscuolo, A. (2011). morePhyML: improving the phylogenetic tree space exploration with PhyML 3. *Molecular Phylogenetics and Evolution* 61(3)**,** 944-948. doi: 10.1016/j.ympev.2011.08.029.

Huson, D.H., and Scornavacca, C. (2012). Dendroscope 3: an interactive tool for rooted phylogenetic trees and networks. *Systematic Biology* 61(6)**,** 1061-1067. doi: 10.1093/sysbio/sys062.

Larkin, M.A., Blackshields, G., Brown, N.P., Chenna, R., McGettigan, P.A., McWilliam, H., et al. (2007). Clustal W and Clustal X version 2.0. *Bioinformatics* 23(21)**,** 2947-2948. doi: 10.1093/bioinformatics/btm404.

Talavera, G., and Castresana, J. (2007). Improvement of phylogenies after removing divergent and ambiguously aligned blocks from protein sequence alignments. *Systematic Biology* 56(4)**,** 564-577. doi: 10.1080/10635150701472164.
